# Primary Exposure to SARS-CoV-2 via Infection or Vaccination Determines Mucosal Antibody-Dependent ACE2 Binding Inhibition

**DOI:** 10.1093/infdis/jiad385

**Published:** 2023-09-07

**Authors:** Janeri Fröberg, Vera J C H Koomen, Christa E van der Gaast-de Jongh, Ria Philipsen, Corine H GeurtsvanKessel, Rory D de Vries, Marije C Baas, Renate G van der Molen, Marien I de Jonge, Luuk B Hilbrands, Martijn A Huynen, Dimitri A Diavatopoulos

**Affiliations:** Department of Laboratory Medicine, Laboratory of Medical Immunology, Radboud University Medical Center, Nijmegen; Radboudumc Center for Infectious Diseases, Radboud University Medical Center, Nijmegen; Department of Laboratory Medicine, Laboratory of Medical Immunology, Radboud University Medical Center, Nijmegen; Radboudumc Center for Infectious Diseases, Radboud University Medical Center, Nijmegen; Department of Nephrology, Radboud University Medical Center, Nijmegen; Department of Laboratory Medicine, Laboratory of Medical Immunology, Radboud University Medical Center, Nijmegen; Radboud Technology Center Clinical Studies, Radboud University Medical Center, Nijmegen; Department of Viroscience, Erasmus Medical Center, Rotterdam; Department of Viroscience, Erasmus Medical Center, Rotterdam; Department of Nephrology, Radboud University Medical Center, Nijmegen; Department of Laboratory Medicine, Laboratory of Medical Immunology, Radboud University Medical Center, Nijmegen; Department of Laboratory Medicine, Laboratory of Medical Immunology, Radboud University Medical Center, Nijmegen; Radboudumc Center for Infectious Diseases, Radboud University Medical Center, Nijmegen; Department of Nephrology, Radboud University Medical Center, Nijmegen; Department of Medical BioSciences, Radboud University Medical Center, Nijmegen, The Netherlands; Department of Laboratory Medicine, Laboratory of Medical Immunology, Radboud University Medical Center, Nijmegen; Radboudumc Center for Infectious Diseases, Radboud University Medical Center, Nijmegen

**Keywords:** ACE2 inhibition, infection, mucosal antibodies, SARS-CoV-2, vaccination

## Abstract

**Background:**

Mucosal antibodies play a critical role in preventing SARS-CoV-2 infections or reinfections by blocking the interaction of the receptor-binding domain (RBD) with the angiotensin-converting enzyme 2 (ACE2) receptor on the cell surface. In this study, we investigated the difference between the mucosal antibody response after primary infection and vaccination.

**Methods:**

We assessed longitudinal changes in the quantity and capacity of nasal antibodies to neutralize the interaction of RBD with the ACE2 receptor using the spike protein and RBD from ancestral SARS-CoV-2 (Wuhan-Hu-1), as well as the RBD from the Delta and Omicron variants.

**Results:**

Significantly higher mucosal IgA concentrations were detected postinfection vs postvaccination, while vaccination induced higher IgG concentrations. However, ACE2-inhibiting activity did not differ between the cohorts. Regarding whether IgA or IgG drove ACE2 inhibition, infection-induced binding inhibition was driven by both isotypes, while postvaccination binding inhibition was mainly driven by IgG.

**Conclusions:**

Our study provides new insights into the relationship between antibody isotypes and neutralization by using a sensitive and high-throughput ACE2 binding inhibition assay. Key differences are highlighted between vaccination and infection at the mucosal level, showing that despite differences in the response quantity, postinfection and postvaccination ACE2 binding inhibition capacity did not differ.

## Teaser

This study compared longitudinal changes in nasal antibody levels and their ACE2-inhibiting activity. Although ACE2-inhibiting activity after primary infection and vaccination was similar, infection-induced ACE2 inhibition depended mostly on mucosal IgA, while post-vaccination inhibition was mainly attributable to mucosal IgG.

The COVID-19 pandemic, caused by the introduction of SARS-CoV-2 in an immunologically naive population, has provided a unique opportunity to study *de novo* immune responses induced by infection or vaccination. Most immunological studies on SARS-CoV-2 to date have focused on serum antibodies and not mucosal antibodies. Mucosal antibodies play a critical role in preventing SARS-CoV-2 infections and reinfections, by blocking the interaction of the receptor-binding domain (RBD) on the viral spike protein with the angiotensin-converting enzyme 2 (ACE2) receptor that is expressed on the surface of host cells [1–4].

Given the key role of mucosal antibodies in providing a first line of defense against infection, improved knowledge about local antibody concentration and function could provide important insights into interrupting SARS-CoV-2 transmission [1, 5, 6]. We and others have shown that SARS-CoV-2 infection generates a strong mucosal antibody response against the spike protein, which is not always correlated to the serum response [5, 7–9], and that early induction of such antibodies is associated with faster symptom resolution and lower viral loads as compared with later development of mucosal antibodies [5, 10]. Several studies have demonstrated that SARS-CoV-2 infection induces mucosal antigen–specific B cells [7, 11, 12], suggesting that mucosal antibodies are produced locally. Although studies have found that COVID-19 vaccination also induces mucosal IgG to the spike protein [11, 13], nasal IgG after parenteral vaccination is likely not due to local production but primarily the result of active transport of serum IgG via the neonatal Fc receptor [11].

The composition of the immune response and its capacity to neutralize SARS-CoV-2 may differ after infection or vaccination. This has not been extensively investigated, largely because mucosal specimens are difficult to analyze in the plaque reduction neutralization test (PRNT) because of lower antibody concentrations as compared with serum. In this study, we compared the mucosal antibody response after primary infection or after primary vaccination with the Spikevax vaccine (mRNA-1273; Moderna). We used a multiplex bead-based approach to assess and compare longitudinal changes in the quantity and neutralizing capacity of mucosal antibodies against ancestral SARS-CoV-2 (Wuhan-Hu-1), as well as Delta and Omicron BA.1 RBD. Moreover, we analyzed and compared how the relationships between antibody concentration and ACE2 inhibition capacity vary between infection and vaccination.

## METHODS

### Cohort Description

To investigate the differences between infection- and vaccination-induced mucosal antibody responses, we used clinical data and samples from 2 cohorts—henceforth, the “infection” and “vaccination” cohorts. All participants signed an informed consent form before participating.

The infection cohort consisted of individuals who participated in the MuCo study (ClinicalTrials.gov NCT04590352) [5]. Conducted during the first COVID-19 wave between March and April 2020, this study included 50 hospital workers with a polymerase chain reaction–confirmed SARS-CoV-2 infection and their household members. In the current analysis, we only included cases who tested positive by polymerase chain reaction at study start (n = 84, 82%) or had a positive serology test result at 28 days after study start (n = 19, 18%). Nasal mucosal lining fluid (MLF) was obtained by nasosorption at study start, at 7 and 28 days, and at 9 months after study start. COVID-19 vaccines were not yet available during this study period.

The vaccination cohort included participants from the RECOVAC immune response study, a prospective and controlled multicenter study designed to investigate the immunogenicity and safety of COVID-19 vaccination in patients with kidney disease or kidney transplantation who received 2 vaccinations between 24 February and 8 April 2021 (ClinicalTrials.gov NCT05030974) [14]. For the current analysis, we included samples from the control group, representing individuals without known kidney disease (n = 46). All participants had no measurable serum antibodies against the nucleocapsid protein (N serology) at study start and at 28 days. One individual had a positive N serology at 6 months and was excluded from analysis at this time point. All participants were vaccinated with 1 dose of Spikevax at study start and again at 28 days after study start. MLF was obtained at study start, 28 days after study start, and at 28 days and 6 months after the second vaccination.

To compare mucosal antibodies between the cohorts, this study focused on the samples collected at study start, at 28 days after infection or second vaccination (+28D), and at the 6- or 9-month follow-up time points.

### MLF Preparation

The method of MLF sampling and elution has been described previously [5]. In short, a nasal sampling device (Nasosorption FX·i; Hunt Developments) was inserted gently into the nose, after which a finger was pressed against the nostril for 60 seconds. MLF strips were frozen immediately after collection and stored at −80 °C until elution for the infection cohort and at −20 °C for the vaccination cohort.

### Antibody Quantification

Antibodies in MLF were quantified with a bead-based multiplex immunoassay (MIA), as described previously [5]. The following antigens were conjugated to Luminex MagPlex beads 02, 24, 60, 28, and 45, respectively: Wuhan-Hu-1 trimeric spike (D614G mutant; ExcellGene), Wuhan-Hu-1 Nucleocapsid-His recombinant (Sino Biologicals), Wuhan-Hu-1 RBD (ExcellGene), Delta RBD (L452R/T478K; Sino Biologicals), and Omicron BA.1 RBD (B.1.1.529/Y508H; Sino Biologicals). MLF samples were thawed, heat-inactivated at 56 °C for 1 hour, and incubated in a dilution of 1:5 with the antigen-coated beads at room temperature. Following a 45-minute incubation, the beads were washed and incubated in a 1:200 dilution with phycoerythrin-conjugated goat anti-human IgG or IgA (Southern Biotech) for 20 minutes and washed again twice. Samples were measured on the Luminex machine with Flexmap 3D and xPONENT software. MFI values were converted to binding antibody units (BAU) by arbitrarily assigning the reference serum (calibrated to the World Health Organization’s International Standard [15]) a starting concentration of 1000 BAU/mL for IgG and IgA. Dilution factors of 3 and 2 for the reference serum were used for IgG and IgA, respectively. Samples were interpolated to the IgA or IgG standard curve with a log 5-parameter logistic regression and log–log axis transformation by BioPlex Manager 6.2 software (Bio-Rad Laboratories), and exported to RStudio.

### ACE2 Binding Competition Assay

To assess the neutralizing capacity of the MLF samples, we established an in-house ACE2 binding competition assay based on the ACE2-RBD assay by Junker et al [16]. The same antigen-conjugated magnetic beads that were used in the MIA were vortexed and sonicated before preparation of a bead solution of 2000 beads/25 μL (8 × 10E4 beads/mL) in assay buffer, with 500 beads/25 μL used per analyte. In a 96-well plate setup, 25 μL of beads were mixed with 25 μL of MLF sample and incubated for 30 minutes at room temperature. Subsequently, 25 μL of human recombinant (His-Tag) biotinylated ACE2 protein dilution (Sino Biological; corresponding to 0.25 mg/mL) was added and incubated for 20 minutes at room temperature. For quality control, 2 wells containing only buffer and 3 wells containing the same quality control sample of pooled serum from individuals with confirmed recent infection in the Omicron era were added to each plate. Based on the assay by den Hartog et al [17], a 10-point dilution of an in-house standard was added to each plate, consisting of pooled serum from infected and vaccinated individuals, and calibrated to the World Health Organization’s International Standard [15]. Samples were measured by the MIA method described previously. A unit of inhibiting arbitrary units per milliliter (IAU/mL) was calculated by the same method as the MIA, and values were exported to RStudio.

To compare the ACE2 binding inhibition assay with the PRNT [18], serum samples were selected from participants in the RECOVAC study that were previously measured by PRNT (n = 74 [14]). These samples were also measured in the ACE2 binding inhibition assay and showed high correlation with the PRNT, with an *R* > 0.9 for all variant combinations except RBD Omicron (*R* = 0.82; Supplementary Figure 1).

### Statistical Analysis

All analyses were performed in RStudio version 2022.02.1 with R software version 4.1.3 [19]. Data wrangling and statistical analyses were performed with the *dplyr*, *tidyr*, *lmer*, and *lme4* packages, and the *ggplot* and *patchwork* packages were used for data visualization. Samples with a value below the lower limit of detection for a particular analyte were manually assigned a value of 0.5 times the lowest measurable value for that analyte. Samples with a value above the limit of detection for a particular analyte were remeasured in a higher dilution (1:80 and 1:1200). When a sample was measured more than once, the mean of the value was used for data analysis.

Differences between paired time points were calculated by the nonparametric two-tailed Friedman test with Dunn posthoc testing and Bonferroni adjustment for multiple testing. Differences between cohorts were calculated with the two-tailed Wilcoxon rank sum test. Correlations were calculated with a Spearman rank correlation. Statistical parameters, such as the sample sizes, measures of distribution, and *P* value thresholds for significance, are reported directly in the figures and figure legends. The significance threshold was set at a corrected *P* value <.05.

To estimate the effect of virus antigen–specific IgA and IgG antibodies on ACE2 binding inhibition over time, we constructed a random intercept mixed-effects model. We specified a separate model per cohort and per variant-antigen combination. IgA and IgG BAU/mL values were separately scaled from 0 to 1 to enable comparison of the isotype-specific effects. Besides antibody concentrations, the model included study day and age as explanatory variables, which were included after univariate analysis showed a significant effect on ACE2 binding inhibition, and participant identification as a random effect.

The formula for the model, in R notation, was as follows:


ACE2bindinginhibition∼IgA_scaled+IgG_scaled+Studyday+Age+(1|ID)

To evaluate the goodness of fit of the model, the predicted ACE2 inhibition activity was plotted, and the Akaike information criterion (AIC) of the final model and univariate models was compared. Furthermore, the normality and variation of the residuals and homoscedasticity of the data were examined with the R *performance* package. Estimates for the covariates, as well as 95% CIs and *P* values (Satterthwaite approximation to degrees of freedom), were extracted and plotted, and the AIC and residual ranges of the final models were reported.

## RESULTS

### Cohort Description

This study includes participants from two cohorts. The infection cohort consisted of 103 confirmed cases with mild SARS-CoV-2 infection. The median age of the infection cohort was 41 years (IQR, 20–52) and 58% was female. A more complete description of the infection cohort was published previously [5]. The vaccination cohort included 46 individuals (59% female). Participants were defined as being noninfected before and during the study by having a serum antibody response against the nucleocapsid protein <19.7 arbitrary units per ml(AU/mL) [17]. The median age of the vaccination cohort was significantly higher than that of the infection cohort (56 years [IQR, 49–65], *P* = .009e-6; Supplementary Figure 2).

### Comparable ACE2 Binding Inhibition Between Vaccinated and Infected Individuals

**Figure 1.**
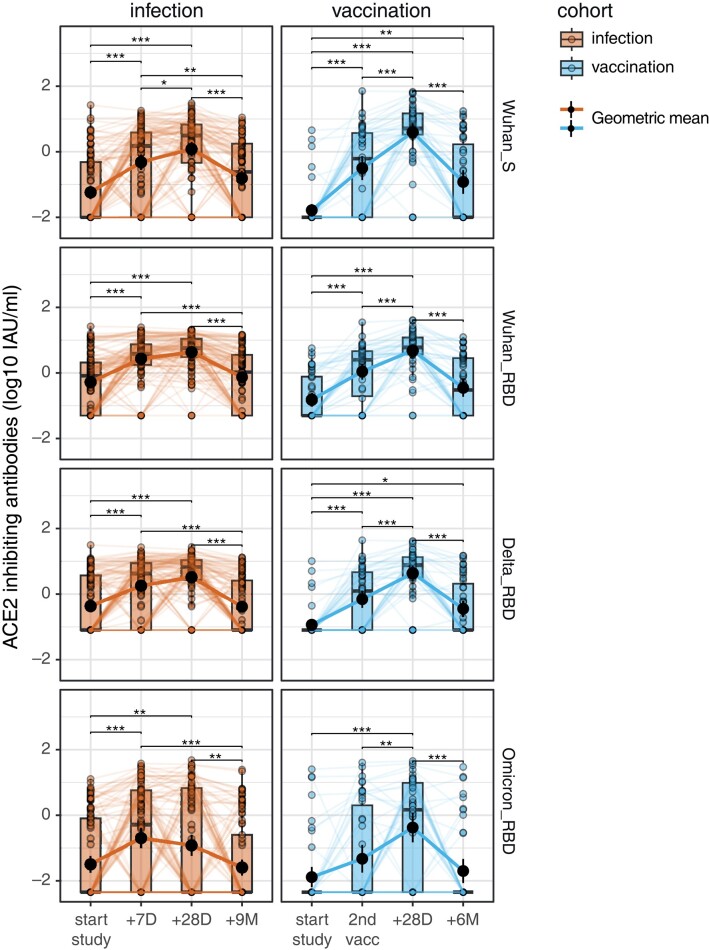
ACE2-inhibiting activity of mucosal antibodies increases shortly after infection and vaccination but wanes over time. ACE2 binding inhibition levels (log_10_ IAU/mL) against the ancestral Wuhan-Hu-1 S and RBD, as well as the Delta and Omicron RBDs, at different time points. Infection cohort: study start and 7 days, 28 days, and 9 months after. Vaccination cohort: study start, moment of second vaccination (28 days after first), and 28 days and 6 months after. The geometric mean of each cohort is depicted as a solid line and black solid dot with the verticle line as the 95% confidence interval. Differences over time are measured by the Friedman test with post hoc Dunn test. *P < .05. **P < .01. ***P < .001. ACE2, angiotensin-converting enzyme 2; IAU, inhibiting arbitrary units; RBD, receptor-binding domain.

To assess the capacity of mucosal antibodies to inhibit binding of viral antigens to ACE2, we measured ACE2 binding inhibition in MLF samples for Wuhan-Hu-1 spike and for Wuhan-Hu-1, Delta, and Omicron RBD. Wuhan-Hu-1 was the circulating virus at the time of the infection study and is incorporated into the Spikevax vaccine. Delta and Omicron were included to analyze the immune response against variants of concern (VOCs). At baseline, the ACE2 binding inhibition was higher in the infection cohort against all but the Omicron variant, as these samples were collected at 2 to 12 days after the onset of symptoms. Consequently, the infection cohort showed more variable increases when compared with the vaccination cohort (Supplementary Figure 3 [5]). Both cohorts had a rapid and significant increase in ACE2 binding inhibition after baseline at the early sampling time points: at 7 days postinfection and at 28 days after the first vaccination dose (Figure 1). ACE2 binding inhibiting activity peaked at 28 days after infection/second vaccination (+28D) and significantly decreased for all viral antigens at the follow-up time point (ie, 6 or 9 months). In the infection cohort at 9 months postinfection, ACE2 binding inhibition was not significantly different anymore from baseline activity for all the viral antigens (*P* = .059 for Wuhan-Hu-1 S). In the vaccination cohort at 6 months after the second vaccination, ACE2 binding inhibition of Wuhan spike (Wuhan-Hu-1 S) and Delta RBD was still significantly elevated as compared with baseline (*P* = .003 and *P* = .022, respectively; Figure 1). The peak ACE2 binding inhibition activity at +28D was not significantly different between the cohorts, except for Wuhan-Hu-1 S, which was higher in the vaccination cohort (*P* = .0025; Supplementary Figure 4*A*). At the follow-up time points, no statistically significant differences were found between the cohorts (Supplementary Figure 4*B*).

### Infection and Vaccination Induce Distinct Mucosal Antibody Responses

**Figure 2.**
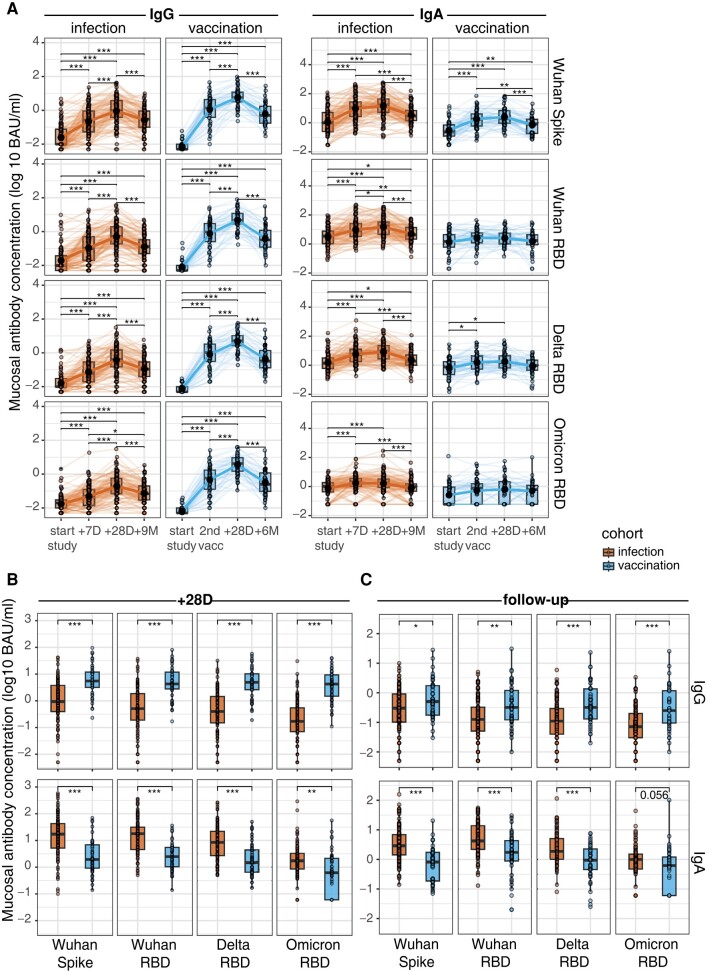
Concentrations of mucosal IgA and IgG depend on exposure type. *A*, Mucosal IgG and IgA concentrations (log_10_ BAU/mL) against the ancestral Wuhan-Hu-1 S and RBD, as well as the Delta and Omicron RBDs, at the various time points. Infection cohort: study start and 7 days, 28 days, and 9 months after. Vaccination cohort: study start, moment of second vaccination (28 days after first), and 28 days and 6 months after. The geometric mean of each cohort is depicted as a solid line and black solid dot with the verticle line as the 95% confidence interval. *B*, Peak IgG and IgA concentrations (28 days) against the ancestral Wuhan-Hu-1 S and RBD and the Delta and Omicron RBDs. *C*, Follow-up IgG and IgA concentrations against the ancestral Wuhan-Hu-1 S and RBD and the Delta and Omicron RBDs: at 9 months for the infection cohort and 6 months for the vaccination cohort. Differences over time are measured with the Friedman test with post hoc Dunn test. Differences between the groups are calculated with the Wilcoxon rank sum test. *P < .05. **P < .01. ***P < .001. BAU, binding antibody units; RBD, receptor-binding domain.

To determine whether mucosal IgA or IgG are induced by SARS-CoV-2 infection or vaccination, we quantified the concentrations of SARS-CoV-2 antigen–specific IgG and IgA in the MLF samples of all participants. Again, we observed higher concentrations of IgG and IgA at study start for the infection cohort (Supplementary Figure 5). IgG generally revealed a similar pattern to the ACE2 binding inhibition capacity. Both cohorts showed a significant increase over baseline against all antigen-variant combinations (Figure 2*A*). Although antibody concentrations in both cohorts significantly waned from 28 days to 6 and 9 months, their mucosal IgG antibody concentrations were significantly higher than baseline for all antigen-variant combinations (*P* < .001). Infection significantly induced IgA antibodies against all antigen-variant combinations, which remained elevated up to 9 months. In contrast, vaccination did not result in significant increases, except for Wuhan-Hu-1 S and Delta RBD (Figure 2*A*). Likewise, although peak IgG concentrations in the vaccination cohort were significantly higher for all variants vs the infection cohort, an opposite pattern was observed for IgA, with higher peak concentrations in the infection cohort (Figure 2*B*). This pattern was maintained at the later time points (Figure 2*C*).

### Type of Primary Exposure Determines Underlying Mechanisms of ACE2 Inhibition

**Figure 3.**
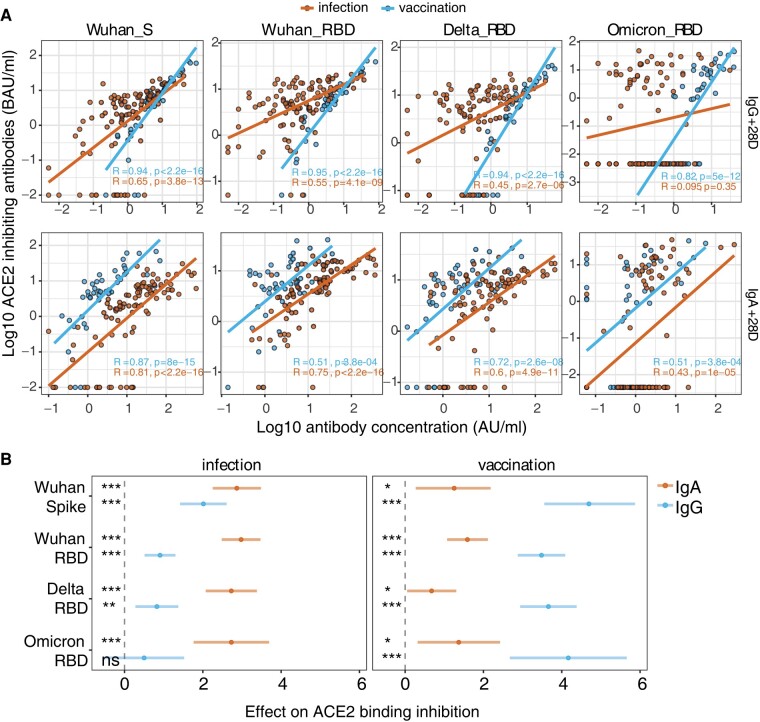
ACE2-inhibiting activity after infection is driven more by IgA, while postvaccination inhibition is mainly IgG driven. *A*, Correlations of IgA and IgG concentrations (log_10_ BAU/mL) and ACE2-inhibiting activity of mucosal antibodies (log_10_ IAU/mL). Spearman correlations were performed, and the *R* and *P* values are depicted for each cohort. *P < .05. **P < .01. ***P < .001. *B*, The predicted change in ACE2 binding inhibition per unit increase of the scaled antibody level is presented with 95% CIs based on the SEM, and two-sided *P* values for the association are plotted on the right, corrected for multiple testing with the Bonferroni method. ACE2, angiotensin-converting enzyme 2; BAU, binding antibody units; IAU, inhibiting arbitrary units.

To investigate the relationship between mucosal antibody concentrations and ACE2 binding inhibition, we correlated the antibody responses with the ACE2 binding inhibition values. For both cohorts, IgG and IgA concentrations showed significant positive correlations with ACE2 binding inhibition. Interestingly, many participants in the infection cohort demonstrated high ACE2 inhibition capacity at low IgG concentrations, suggesting that other antibody classes contribute to neutralization. Indeed, in the infection cohort, ACE2 inhibition correlated stronger with IgA than with IgG (*R* = 0.09–0.65 for IgG and *R* = 0.40–0.81 for IgA; Figure 3*A*). Vaccination-induced ACE2 binding inhibition was most strongly correlated with IgG (*R* = 0.82–0.95 for IgG and *R* = 0.51–0.87 for IgA; Figure 3*A*).

We know that other factors contribute to the variation in ACE2 binding inhibition, such as time since exposure and age of the participant. To assess the contribution of antibody isotype to ACE2 inhibition while correcting for the influence of age and time, we performed a linear mixed-effects model per cohort and variant. In this model, ACE2 inhibition was the outcome, and study day, age, and the scaled IgA and IgG concentrations were covariates. The predictions from this linear model fitted well with the observed data, although the out-of-range values were not predicted as well and increased the AIC (Supplementary Figure 6*A* and 6*B* and Supplementary Table 1). We observed that for both cohorts the quantity of IgG and IgA had a significant and positive effect on the ACE2-inhibiting capacity. After infection, mainly IgA contributes to ACE2 inhibition. This IgA dominance becomes more prominent against the VOCs, with IgA having a larger effect on ACE2 inhibition than IgG in Delta and Omicron (Figure 3*B*). Conversely, ACE2 inhibition was mostly driven by IgG in the vaccination cohort, against the ancestral variant and the VOCs.

## DISCUSSION

Blocking the interaction of SARS-CoV-2 with the ACE2 receptor is an essential function of mucosal antibodies to prevent or reduce SARS-CoV-2 infection and replication [1, 20, 21]. Differences in functionality between infection- and vaccination-induced mucosal antibodies have not been extensively investigated. We compared the mucosal antibody response after primary infection and primary vaccination. Although ACE2-inhibiting activity did not differ between the infection and vaccination cohorts, we found that infection induced higher mucosal IgA at 28 days and at 6 or 9 months. Importantly, we show that how mucosal antibodies neutralize SARS-CoV-2 differs by the type of primary exposure. After correcting for age and time points, infection-induced ACE2 binding inhibition was mainly driven by mucosal IgA, while postvaccination this was mostly mucosal IgG.

Our cohorts consisted of individuals exposed to the Wuhan-Hu-1 strain by vaccination or by infection. Consequently, we observed lower responses toward the Omicron variant, similar to previous data [22–26]. The vaccination cohort demonstrated higher inhibition against the Wuhan spike protein at day 28 than the infection cohort, in line with previous publications [27, 28]. No differences between the infection and vaccination cohorts were found with regard to ACE2 binding inhibition against the other VOCs. An explanation for the lower ACE2-inhibiting activity postinfection for the Wuhan spike may be that, as an effect of viral load and duration of infection, there is a more variable level of antigen exposure postinfection. In contrast, vaccination is performed with a standard human dose of mRNA.

Although we show that infection and vaccination induce mucosal IgA, the IgA concentrations after infection were significantly higher than after vaccination, especially at the follow-up time point, which was striking considering that the postinfection samples were collected 3 months later than the postvaccination samples. Furthermore, increased IgA concentrations postvaccination were found only against the Wuhan-Hu-1 spike protein. This is not unexpected, as it is known that intramuscular vaccination mainly elicits an IgG response [29, 30] and IgA is generally not effectively transported to the mucosal surface [6, 11, 31].

For IgG, we found that the infection cohort had less significant and more variable responses over baseline than the vaccination cohort. This could partly be due to the cohort selection, as some participants of the infection cohort were not yet infected at study start while others had already been infected for approximately 6 days at study start (Supplementary Figure 3). The antibody response will therefore not be completely synchronized for the infection cohort as opposed to the vaccination cohort.

The observation that infection induces a broader antibody profile than vaccination has been described [32–35]. In the case of parenteral influenza vaccines, vaccination induces an IgG response but fails to induce mucosal IgA responses, while infection gives rise to IgG and IgA formation [33]. A study examining memory B-cell responses after SARS-CoV-2 infection and vaccination found that the memory B cells after infection evolved for a longer period, resulting in greater potency and breadth than the vaccine-induced memory B cells [36].

In this study, we used an ACE2 binding inhibition assay to analyze MLF, following a similar approach [16]. By including a dilution series of an external standard on each plate and fitting the results to the standard curve, we were able to account for batch and dilution effects (Supplementary Figure 7*A* and 7*B*). However, we recognize that the sensitivity of the ACE2 binding inhibition assay on MLF as compared with the MIA is lower, resulting in some out-of-range samples that were given an arbitrary low value (Figure 3*A*). We chose to include these samples in the correlation assay and modeling to account for the limitations of the measurement. Overall trends in the correlation and modeling analyses stayed the same when we performed a sensitivity analysis with only the in-range values, although confidence intervals increased due to a significantly lower sample size. Our assay showed high correlation with the PRNT: the gold standard for measuring neutralizing capacity in serum (Supplementary Figure 1). The ACE2 binding inhibition assay is not dependent on cell culture, and because of the bead-based approach, additional variants can be easily added. Moreover, we demonstrate that the assay, though a bit less sensitive, is able to detect changes in functional mucosal antibodies, making it an attractive method for clinical or epidemiological studies.

This study has several limitations. First, because of the use of two cohorts from different studies, age and sampling time points did not completely match. The vaccination cohort was significantly older than the infection cohort, mostly due to the fact that the infection cohort included children. However, in multivariable analysis, the effect of age did not remain significant in any of the models, so we do not consider this to be an issue in the final model. Second, the late follow-up time points for the two cohorts differed, precluding direct comparison between the cohorts. We thus cannot exclude that the observations at this time point are the result of differences in timing. Yet, the IgA concentrations in the infection cohort were higher at 9 months than the IgA concentrations in the vaccination cohort were at 6 months, thus most likely representing a true biological effect. Finally, we recognize that antibodies do not tell the complete story of protection. We did not analyze memory T- and B-cell responses or other antibody functionalities, which could also play an important role in protection [37, 38].

In conclusion, we have analyzed the mucosal antibody response to primary exposure to SARS-CoV-2 as a result of infection or Spikevax vaccination. Our study found that although the ACE2 binding inhibition capacities in both cohorts were similar, it was dependent on different antibody isotypes.

## Supplementary Data

Supplementary materials are available at *The Journal of Infectious Diseases* online. Consisting of data provided by the authors to benefit the reader, the posted materials are not copyedited and are the sole responsibility of the authors, so questions or comments should be addressed to the corresponding author.

## Supplementary Material

jiad385_Supplementary_Data

## Notes

***Acknowledgments.*** We thank all MuCo and RECOVAC study participants for their willingness to participate and their support for the study. Moreover, we thank J. F. Sanders, D. van Baarle, A. L. Messchendorp, C. C. Baan, F. J. Bemelman, M. E. J. Reinders, G. den Hartog, R. van Binnendijk, R. T. Gansevoort, M. M. L. Kho, and E. B. M. Remmerswaal of the RECOVAC consortium for their help and support. Special thanks to Fred van Opzeeland, Liana Barenbrug, Joyce Rust, and Wouter Mattheussens for their contribution to the experimental work and study logistics.

***Author contributions.*** Conceptualization: D. A. D., M. A. H., R. G. M., M. C. B., R. P., and L. B. H. Laboratory processing: J. F., V. J. C. H. K., and C. E. G.-J. Formal analysis: J.F. and V. J. C. H. K. Writing–original draft preparation: J. F., and V. J. C. H. K. Writing–review and editing: J. F., V. J. C. H. K., D. A. D., M. A. H., L. B. H., R. G. M., R. D. V., C. H. G.K., and M. I. J. Resources: C. H. G.K. and R. D. V. Supervision: D. A. D., M. A. H., and L. B. H. J. F. and V. J. C. H. K. contributed equally to this manuscript. M. A. H. and L. B. H. contributed equally to this manuscript.

***Data availability.*** The raw data are available from the corresponding author upon reasonable request. The raw data are not publicly available due to data and patient's privacy laws. Source data are provided with this article.

***Code availability.*** The R code that supports the findings of this study is available from the corresponding author upon reasonable request. The raw code is not publicly available due to data and patient's privacy laws.

***Financial support*.** The MuCo study was funded by the Laboratory of Medical Immunology, Radboud University Medical Center, and the RECOVAC consortium was funded by ZonMW (project number: 10430072010002) and the Nierstichting. Funding to pay the Open Access publication charges for this article was provided by ZonMW.

## References

[1] Froberg J , Diavatopoulos DA. Mucosal immunity to severe acute respiratory syndrome coronavirus 2 infection. Curr Opin Infect Dis 2021; 34:181–6. 33899752 10.1097/QCO.0000000000000724

[2] Hoffmann M , Kleine-Weber H, Schroeder S, et al SARS-CoV-2 cell entry depends on ACE2 and TMPRSS2 and is blocked by a clinically proven protease inhibitor. Cell 2020; 181:271–80.e8. 32142651 10.1016/j.cell.2020.02.052 PMC7102627

[3] Shang J , Wan Y, Luo C, et al Cell entry mechanisms of SARS-CoV-2. PNAS 2020; 117:11727–34. 32376634 10.1073/pnas.2003138117 PMC7260975

[4] Khatri I , Staal FJ, Van Dongen JJ. Blocking of the high-affinity interaction-synapse between SARS-CoV-2 spike and human ACE2 proteins likely requires multiple high-affinity antibodies: an immune perspective. Front Immunol 2020; 11:570018. 33042151 10.3389/fimmu.2020.570018 PMC7527437

[5] Fröberg J , Gillard J, Philipsen R, et al SARS-CoV-2 mucosal antibody development and persistence and their relation to viral load and COVID-19 symptoms. Nat Commun 2021; 12:5621. 34556667 10.1038/s41467-021-25949-x PMC8460778

[6] Russell MW , Moldoveanu Z, Ogra PL, Mestecky J. Mucosal immunity in COVID-19: a neglected but critical aspect of SARS-CoV-2 infection. Front Immunol 2020; 11:611337. 33329607 10.3389/fimmu.2020.611337 PMC7733922

[7] Roltgen K , Boyd SD. Antibody and B cell responses to SARS-CoV-2 infection and vaccination. Cell Host Microbe 2021; 29:1063–75. 34174992 10.1016/j.chom.2021.06.009 PMC8233571

[8] Ravichandran S , Grubbs G, Tang J, et al Systemic and mucosal immune profiling in asymptomatic and symptomatic SARS-CoV-2–infected individuals reveal unlinked immune signatures. Sci Adv 2021; 7:eabi6533. 34644111 10.1126/sciadv.abi6533 PMC8514093

[9] Wright PF , Prevost-Reilly AC, Natarajan H, et al Longitudinal systemic and mucosal immune responses to SARS-CoV-2 infection. J Infect Dis 2022; 226:1204–14. 35188974 10.1093/infdis/jiac065 PMC8903457

[10] Callow KA . Effect of specific humoral immunity and some non-specific factors on resistance of volunteers to respiratory coronavirus infection. J Hyg (Lond) 1985; 95:173–89. 2991366 10.1017/s0022172400062410 PMC2129501

[11] Tang J , Zeng C, Cox TM, et al Respiratory mucosal immunity against SARS-CoV-2 after mRNA vaccination. Sci Immunol 2022; 7:eadd4853. 35857583 10.1126/sciimmunol.add4853 PMC9348751

[12] Laidlaw BJ , Ellebedy AH. The germinal centre B cell response to SARS-CoV-2. Nat Rev Immunol 2022; 22:7–18. 34873279 10.1038/s41577-021-00657-1 PMC8647067

[13] Guerrieri M , Francavilla B, Fiorelli D, et al Nasal and salivary mucosal humoral immune response elicited by mRNA BNT162b2 COVID-19 vaccine compared to SARS-CoV-2 natural infection. Vaccines (Basel) 2021; 9:1499. 34960244 10.3390/vaccines9121499 PMC8708818

[14] Sanders JF , Bemelman FJ, Messchendorp AL, et al The RECOVAC immune-response study: the immunogenicity, tolerability, and safety of COVID-19 vaccination in patients with chronic kidney disease, on dialysis, or living with a kidney transplant. Transplantation 2022; 106:821–34. 34753894 10.1097/TP.0000000000003983 PMC8942603

[15] World Health Organization . First WHO International Standard for anti-SARS-CoV-2 immunoglobulin (human). 2020. https://www.nibsc.org/documents/ifu/20-136.pdf. Accessed 17 January 2022.

[16] Junker D , Dulovic A, Becker M, et al COVID-19 patient serum less potently inhibits ACE2-RBD binding for various SARS-CoV-2 RBD mutants. Sci Rep 2022; 12:7168. 35505068 10.1038/s41598-022-10987-2 PMC9062870

[17] den Hartog G , Schepp RM, Kuijer M, et al SARS-CoV-2–specific antibody detection for seroepidemiology: a multiplex analysis approach accounting for accurate seroprevalence. J Infect Dis 2020; 222:1452–61. 32766833 10.1093/infdis/jiaa479 PMC7454740

[18] Okba NM , Müller MA, Li W, et al Severe acute respiratory syndrome coronavirus 2–specific antibody responses in coronavirus disease patients. Emerg Infect Dis 2020; 26:1478. 32267220 10.3201/eid2607.200841 PMC7323511

[19] R Core Team . R: a language and environment for statistical computing. R Foundation for Statistical Computing,Vienna, Austria. 2013. https://www.R-project.org/.

[20] Jiang S , Zhang X, Yang Y, Hotez PJ, Du L. Neutralizing antibodies for the treatment of COVID-19. Nat Biomed Eng 2020; 4:1134–9. 33293725 10.1038/s41551-020-00660-2 PMC7891858

[21] Shi R , Shan C, Duan X, et al A human neutralizing antibody targets the receptor-binding site of SARS-CoV-2. Nature 2020; 584:120–4. 32454512 10.1038/s41586-020-2381-y

[22] Toh ZQ , Mazarakis N, Nguyen J, et al Comparison of antibody responses to SARS-CoV-2 variants in Australian children. Nat Commun 2022; 13:7185. 36434068 10.1038/s41467-022-34983-2 PMC9700848

[23] Cheng SMS , Mok CKP, Leung YWY, et al Neutralizing antibodies against the SARS-CoV-2 Omicron variant BA.1 following homologous and heterologous CoronaVac or BNT162b2 vaccination. Nat Med 2022; 28:486–9. 35051989 10.1038/s41591-022-01704-7 PMC8940714

[24] Qu P , Faraone JN, Evans JP, et al Enhanced evasion of neutralizing antibody response by Omicron XBB.1.5, CH.1.1, and CA.3.1 variants. Cell Rep 2023; 42:112443. 37104089 10.1016/j.celrep.2023.112443 PMC10279473

[25] Dejnirattisai W , Shaw RH, Supasa P, et al Reduced neutralisation of SARS-CoV-2 omicron B.1.1.529 variant by post-immunisation serum. Lancet 2022; 399:234–6. 34942101 10.1016/S0140-6736(21)02844-0 PMC8687667

[26] Pajon R , Doria-Rose NA, Shen X, et al SARS-CoV-2 Omicron variant neutralization after mRNA-1273 booster vaccination. N Engl J Med 2022; 386:1088–91. 35081298 10.1056/NEJMc2119912 PMC8809504

[27] Bekliz M , Adea K, Vetter P, et al Neutralization capacity of antibodies elicited through homologous or heterologous infection or vaccination against SARS-CoV-2 VOCs. Nat Commun 2022; 13:3840. 35787633 10.1038/s41467-022-31556-1 PMC9253337

[28] Hojjat Jodaylami M , Djaileb A, Ricard P, et al Cross-reactivity of antibodies from non-hospitalized COVID-19 positive individuals against the native, B.1.351, B.1.617.2, and P.1 SARS-CoV-2 spike proteins. Sci Rep 2021; 11:21601. 34750399 10.1038/s41598-021-00844-z PMC8575961

[29] Durrer P , Gluck U, Spyr C, et al Mucosal antibody response induced with a nasal virosome-based influenza vaccine. Vaccine 2003; 21(27–30):4328–34. 14505915 10.1016/s0264-410x(03)00457-2

[30] Brokstad KA , Eriksson JC, Cox RJ, et al Parenteral vaccination against influenza does not induce a local antigen-specific immune response in the nasal mucosa. J Infect Dis 2002; 185:878–84. 11920311 10.1086/339710

[31] Horton RE , Vidarsson G. Antibodies and their receptors: different potential roles in mucosal defense. Front Immunol 2013; 4:200. 23882268 10.3389/fimmu.2013.00200 PMC3712224

[32] Nachbagauer R , Choi A, Hirsh A, et al Defining the antibody cross-reactome directed against the influenza virus surface glycoproteins. Nat Immunol 2017; 18:464–73. 28192418 10.1038/ni.3684 PMC5360498

[33] Krammer F . The human antibody response to influenza A virus infection and vaccination. Nat Rev Immunol 2019; 19:383–97. 30837674 10.1038/s41577-019-0143-6

[34] Meyers J , Windau A, Schmotzer C, et al SARS-CoV-2 antibody profile of naturally infected and vaccinated individuals detected using qualitative, semi-quantitative and multiplex immunoassays. Diagn Microbiol Infect Dis 2022; 104:115803. 36162282 10.1016/j.diagmicrobio.2022.115803 PMC9420072

[35] Assis R , Jain A, Nakajima R, et al Distinct SARS-CoV-2 antibody reactivity patterns elicited by natural infection and mRNA vaccination. NPJ Vaccines 2021; 6:132. 34737318 10.1038/s41541-021-00396-3 PMC8568980

[36] Cho A , Muecksch F, Schaefer-Babajew D, et al Anti-SARS-CoV-2 receptor-binding domain antibody evolution after mRNA vaccination. Nature 2021; 600:517–22. 34619745 10.1038/s41586-021-04060-7 PMC8674133

[37] Cox RJ , Brokstad KA. Not just antibodies: B cells and T cells mediate immunity to COVID-19. Nat Rev Immunol 2020; 20:581–2. 32839569 10.1038/s41577-020-00436-4 PMC7443809

[38] Moss P . The T cell immune response against SARS-CoV-2. Nat Immunol 2022; 23:186–93. 35105982 10.1038/s41590-021-01122-w

