# Supplementary material for: Primary Exposure to SARS-CoV-2 via Infection or Vaccination Determines Mucosal Antibody-Dependent ACE2 Binding Inhibition
Source: J Infect Dis. 2023 Sep 7;229(1):137–46. doi: 10.1093/infdis/jiad385 (PMC10786246; doi:10.1093/infdis/jiad385)
Supplement: jiad385_Supplementary_Data [file jiad385_supplementary_data.zip › Table_S1.docx]

| **Model** |  | **Scaled residuals** | | |
| --- | --- | --- | --- | --- |
| Variant_antigen_cohort | **AIC** | min | median | max |
| Wuhan_S_Infection | 679 | -2.870 | 0.166 | 2.455 |
| Wuhan_RBD_ Infection | 518 | -3.281 | 0.101 | 2.786 |
| Delta_RBD_ Infection | 621 | -2.460 | 0.042 | 2.088 |
| Omicron_RBD_ Infection | 979 | -1.860 | -0.171 | 2.234 |
| Wuhan_S_Vaccination | 277 | -3.000 | 0.076 | 3.254 |
| Wuhan_RBD_ Vaccination | 176 | -2.830 | -0.073 | 2.836 |
| Delta_RBD_ Vaccination | 200 | -2.624 | 0.066 | 3.105 |
| Omicron_RBD_ Vaccination | 411 | -2.362 | -0.095 | 2.908 |

**Table S1: Mixed effect model diagnostics.** For each model, the AIC and the range of the scaled residuals is provided.
