# Supplementary material for: Primary Exposure to SARS-CoV-2 via Infection or Vaccination Determines Mucosal Antibody-Dependent ACE2 Binding Inhibition
Source: J Infect Dis. 2023 Sep 7;229(1):137–46. doi: 10.1093/infdis/jiad385 (PMC10786246; doi:10.1093/infdis/jiad385)
Supplement: jiad385_Supplementary_Data [file jiad385_supplementary_data.zip › 20230830_supplementarylegends.docx]

**Figure S1: Correlation of ACE2 competition assay with plaque reduction neutralization test (PRNT) is high.** Log10-transformed PRNT results (y-axis) of serum samples from 74 individuals from the vaccination cohort were correlated against the log10-transformed ACE2 inhibition results of serum of those same individuals (x-axis), for the ancestral Wuhan-Hu-1 S and RBD, and Delta and Omicron RBD. Each dot represents an individual. Spearman correlations were performed, and the R and p-value are depicted in the left upper corner. A linear model was fitted to the data (blue solid line), as well as a x=y line (dashed grey line), and the PRNT cut-off (solid horizonal line).

**Figure S2: The vaccination cohort was older than the infection cohort.** Boxplot and whiskers of the age in years of the infection and the vaccination cohort at time of study start. Differences between the cohorts was calculated using the Wilcoxon rank sum test and exact p-value is depicted in the figure.

**Figure S3: ACE2 inhibiting activity of mucosal antibodies at study start were higher in the infection cohort than the vaccination cohort.** Log10-transformed mucosal ACE2 binding inhibiting antibodies at study start. Results are plotted against the ancestral Wuhan-Hu-1 S and RBD, and Delta and Omicron RBD. Differences between the groups are calculated using the Wilcoxon rank sum test. Orange: infection cohort, blue: vaccination cohort. P-values *p <0.05, **p<0.01, *** p<0.001.

**Figure S4: No differences in mucosal ACE2 binding inhibition (log10 BAU/mL) between infection and vaccination cohort. a.)** Differences of log10-transformed ACE2 binding inhibition levels at the peak timepoint (infection: +28 days post study start, vaccination: +28 days post second vaccination). **b.)** Differences of log10-transformed ACE2 binding inhibition levels at the follow up timepoint (infection: +9 months post study start, vaccination: +6 months post second vaccination. Differences between the groups are calculated using the Wilcoxon rank sum test. Orange: infection cohort, blue: vaccination cohort. P-values *p <0.05, **p<0.01, *** p<0.001.

**Figure S5: Mucosal IgG and IgA concentrations of the infection cohort were higher than the vaccination cohort at study start.** Log10-transformed mucosal IgG and IgA concentrations at study start. Results are plotted against the ancestral Wuhan-Hu-1 S and RBD, and Delta and Omicron RBD. Differences between the groups are calculated using the Wilcoxon rank sum test. Orange: infection cohort, blue: vaccination cohort. P-values *p <0.05, **p<0.01, *** p<0.001.

**Figure S6: Mixed effects model is able to predict ACE2 binding inhibition capacity but does not predict out of range values. a.)** Measured log10 transformed ACE2 binding inhibition for Wuhan-Hu-1_RBD (log10 IAU/mL) at the three timepoints (infection cohort: start study, +28 days and +9 months post study start; vaccination cohort: start study, +28 days, and +6 months post second vaccination). **b.)** Predicted ACE2 binding inhibition for Wuhan-Hu-1_RBD, using the full model (with addition of the IgA and IgG antibody concentrations). Orange: infection cohort, blue: vaccination cohort.

**Figure S7: Effect of sample dilution on the ACE2 result is diminished when using the interpolated concentration. a.)** The ACE2 inhibition results of a set of 74 serum samples in % inhibition. **b.)** The ACE2 inhibition results of a set of 74 serum samples in interpolated concentration (log10 IAU/mL). Each line connects the same sample, measured in three different dilutions (1:100, 1:500, and 1:800), and the box and whiskers represent the median and IQR of the results. W.S: Wuhan-Hu-1_S, W.RBD: Wuhan-Hu-1_RBD, D.RBD: Delta-RBD, O.RBD: Omicron-RBD.
